# Characterization of spinal cord damage based on automatic video analysis of froglet swimming

**DOI:** 10.1242/bio.042960

**Published:** 2019-12-24

**Authors:** Sebastián De Vidts, Emilio Méndez-Olivos, Miriam Palacios, Juan Larraín, Domingo Mery

**Affiliations:** 1 Department of Computer Science, School of Engineering, P. Universidad Católica de Chile; 2 Center for Aging and Regeneration, Facultad de Ciencias Biológicas, P. Universidad Católica de Chile, Santiago de Chile, Chile 7820436

**Keywords:** Froglets, Tracking, Swimming, Video processing

## Abstract

*Xenopus laevis* frogs are a widely used organism to study aspects of modern biology (Harland and Grainger, 2011). Its central nervous system is particularly interesting, because in certain stages of metamorphosis the spinal cord can regenerate after injury and recover swimming. With this in mind, automatic gait analysis could help evaluate the regenerative performance by means of a method that automatically and quantitatively establishes the degree in froglets' limb movement. Here, we present an algorithm that characterizes spinal cord damage in froglets. The proposed method tracks the position of the limbs throughout videos and extracts kinematic features, which posteriorly serve to differentiate froglets with different levels of damage to the spinal cord. The detection algorithm and kinematic features chosen were validated in a pattern recognition experiment in which 90 videos (divided equally in three classes: uninjured, hemisected and transected) were classified. We conclude that our system is effective in the characterization of damage to the spinal cord through video analysis of a swimming froglet with a 97% accuracy. These results potentially validate this methodology to automatically compare the recovery of spinal cord function after different treatments without the need to manually process videos. In addition, the procedure could be used to measure the kinematics and behavioral response of froglets to different experimental conditions such as nutritional state, stress, genetic background and age.

## Abstract (precis)

**Summary:** Automatic video tracking system to measure froglet swimming and characterize the degree of spinal cord damage after spinal cord injury. It can potentially be used to compare the recovery of spinal cord function after different treatments.

## INTRODUCTION

*Xenopus* offers multiple advantages as a model organism to study development, regeneration, behavior and evolution. Some of the advantages it presents for experimentation are: (1) it is easy to breed and generate hundreds of animals developing synchronously at a low cost compared to rodents; (2) the availability of genomic resources and methods to study gene function and availability of genomic resources, and (3) the possibility to perform tissue transplantation (Harland and Grainger, 2011). *Xenopus* frogs are particularly attractive to study central nervous system (CNS) regeneration because they swim at certain developmental stages when they are able to regenerate the spinal cord after injury (SCI) and recover the ability to swim. This ability is progressively lost throughout metamorphosis resulting in non-regenerative froglets and adult frogs that are no longer able to recover after SCI (Gaete et al., 2012; Muñoz et al., 2015; Edwards-Faret et al., 2017). The response to SCI in froglets has shown similar molecular, cellular and clinical hallmarks compared to other animals such as rodents and humans (Lee-Liu et al., 2014; Lee-Liu et al.; 2017), making this a useful model organism to use in studies that could be informative in understanding SCI in humans. In addition, it is quite simple to achieve complex locomotor conditions like paraplegia (with both posterior limbs paralyzed), or hemiplegia (just one posterior limb is paralyzed); two types of lesions that are also observed in humans.

SCI in mammals, including humans, leads to permanent paralysis (paraplegia and quadriplegia) including loss of bowel, bladder and sexual functions, chronic pain and autonomic dysreflexia, among other symptoms. Currently, no efficient treatment for spinal cord regeneration (SCR) has been established; however, using *Xenopus* as a model organism could be of great help for this. The development of a method that can measure froglets' movement could quantify the effects of new treatments and the recovery of injured animals. In addition, this method could be used to study other behaviors in this classic model organism. For the development of this method, kinematics analysis emerges as an invaluable tool. Gait analysis has been used to study many species, including horses (Barrey, 1999), elephants (Hutchinson et al., 2006) and hummingbirds (Warrick et al., 2005). Gait analysis allows the evaluation of pathological conditions in humans (Abbass and Abdulrahman, 2014) as well as in other animals (Swanson et al., 1998; Roxana et al., 2007). The swimming of tadpoles and froglets has been analyzed before with kinematic approaches in order to understand the transition from an axial-based swimming to a limbed propulsion (Combes et al., 2004). These approaches have been done for total distance covered or kinematics analysis by drawing the body outline of the animal, making this method very inefficient and laborious, and also not able to measure more complex parameters such as coordination, or compare slight changes in movement in different experimental conditions.

The benefits of using automatic gait analysis for understanding SCR are analysis of a larger number of videos and during longer periods of time, less error than manual tracking and automatic tracking of all limbs simultaneously. In this way, a model-based visual tracking algorithm has been developed for zebrafish (Fontaine et al., 2008) and fruit fly (Fontaine et al., 2009), both of which achieved a more in-depth understanding of the animal's movement. For example, subtle differences in swimming between normal and mutant zebrafish were measured. An advantage of processing large amounts of videos is the ability to extract efficient features that summarize the information. Features can then be used to compare one or more videos. By using pattern recognition techniques, it is possible to determine which features discriminate best between two or more behaviors.

Here we present an algorithm that analyzes swimming videos and automatically characterizes the level of damage to the spinal cord of a *Xenopus laevis* froglet. From a dorsal view it measures the position of each limb every frame and then analyzes their movement throughout the video. This information is then summarized into four kinematic features that are validated using pattern recognition in a classification experiment. Even though both fore- and hindlimbs are tracked by the algorithm, the analysis was centered on the latter (Combes et al., 2004). We first describe the detection algorithm for the froglet and its limbs. Subsequently, we define four kinematic features derived from expected behaviors. And finally, we validate the ability of the program to classify 90 videos into three levels of spinal cord damage: uninjured, hemisected and transected animals. The development of an algorithm as a tool with discriminative or ‘diagnostic’ capacity could be of great aid to screen for drugs that have potential beneficial effects on SCR. Here we trained the algorithm with previously well-known conditions using training data, and we showed that after learning the algorithm is able to classify blindly between different groups. This algorithm is a great improvement in quantitatively assessing any improvement in SCR after a given treatment. This algorithm would allow the detection of slight improvements in swimming recovery compared to the current methods developed in our own lab that only allow the measurement of the distance swum by froglets (Gaete et al., 2012; Muñoz et al., 2015); a parameter that is too broad to allow the detection of small effects when screening for potential new treatments.

## RESULTS

### Kinematic features

**Fig. 1.**
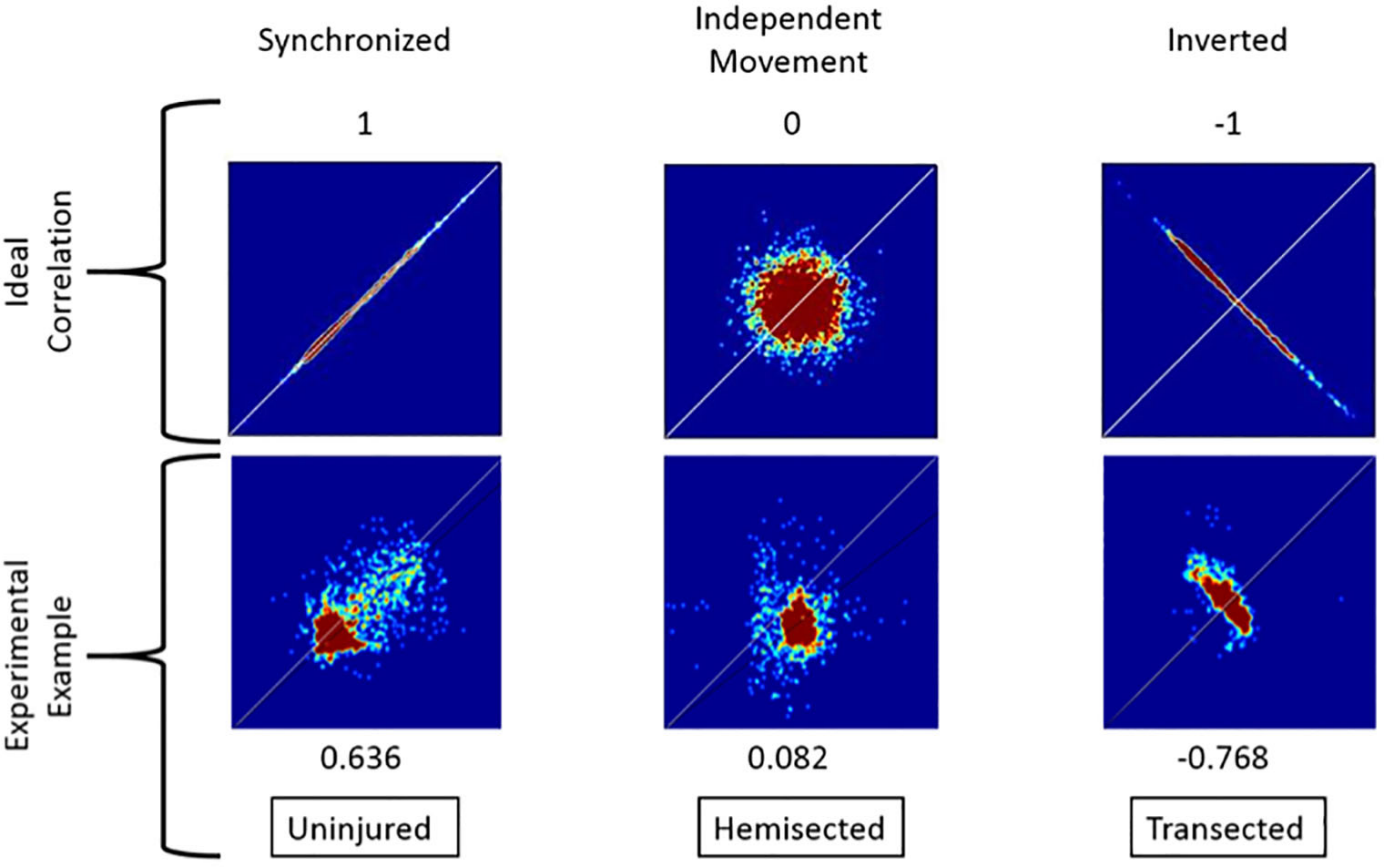
**Synchronization variance in 2D heatmaps and the expected output of each behavior.** An example is given of each type of froglet compared to the ideal correlation of each of the three types of synchronization, previously defined as: synchronized, independent and inverted. Rows show: type of movement, ideal correlation, ideal distribution, one experimental example, measured correlation and original class, respectively.

**Fig. 2.**
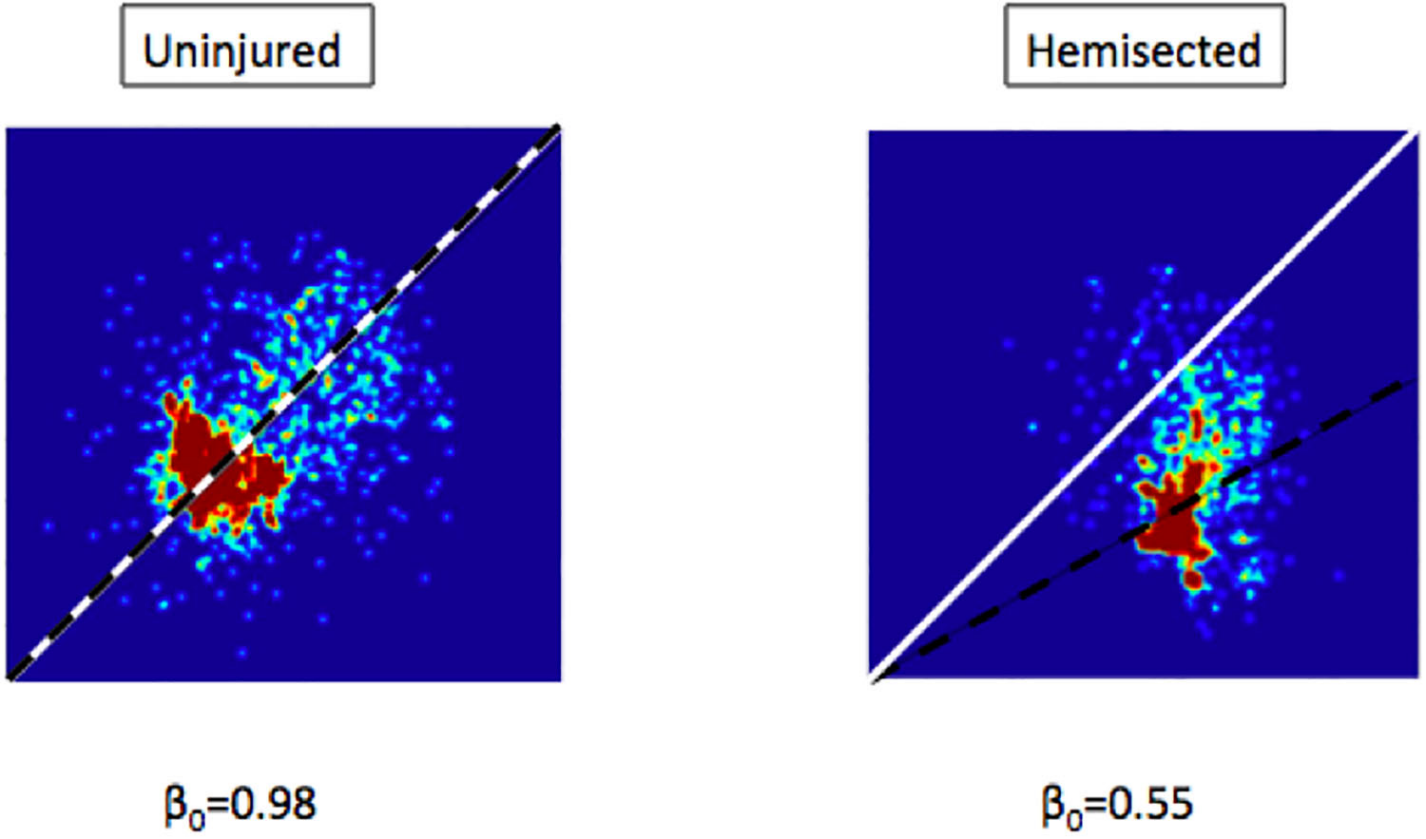
**Symmetry variance in 2D heatmaps.** Two experimental examples illustrate how a difference in symmetry is reflected in the heatmaps. The white line shows perfect symmetry, while the black-dashed line shows the corresponding line of the estimated slope β_0_. We can see that in the healthy froglet both black and white lines are almost the same, giving a β_0_ almost equal to 1. On the other hand, in the hemisected froglet the right foot tends to move approximately half the distance of the left foot's stroke, making white and black lines differ significantly, thus showing a lack of symmetry.

For each video processed, a heatmap of the angles for the right and left hindlimb were obtained and plotted in a 2D histogram (Fig. 1 and 2). In the example of Fig. 1 an uninjured froglet shows a positive correlation with the algorithm (0.636), instead of a negative one for transected swimming (−0.768). So uninjured froglets are detected by displaying synchronized swimming, according to the swimming description made by Combes et al. (2004). Meanwhile in the example of a hemisected froglet in same figure, the correlation is near to 0 (0.082), indicating an independent or non-synchronized movement. On the other hand, examples of symmetry of swimming are shown in Fig. 2. There, an uninjured froglet has a coefficient equal to 0.98, close to 1, but for a hemisected froglet the coefficient is equal to 0.55; indicating a lack of symmetry because of the reduced stroke movement of the right hindlimb (which was damaged) compared to the left hindlimb.

**Fig. 3.**
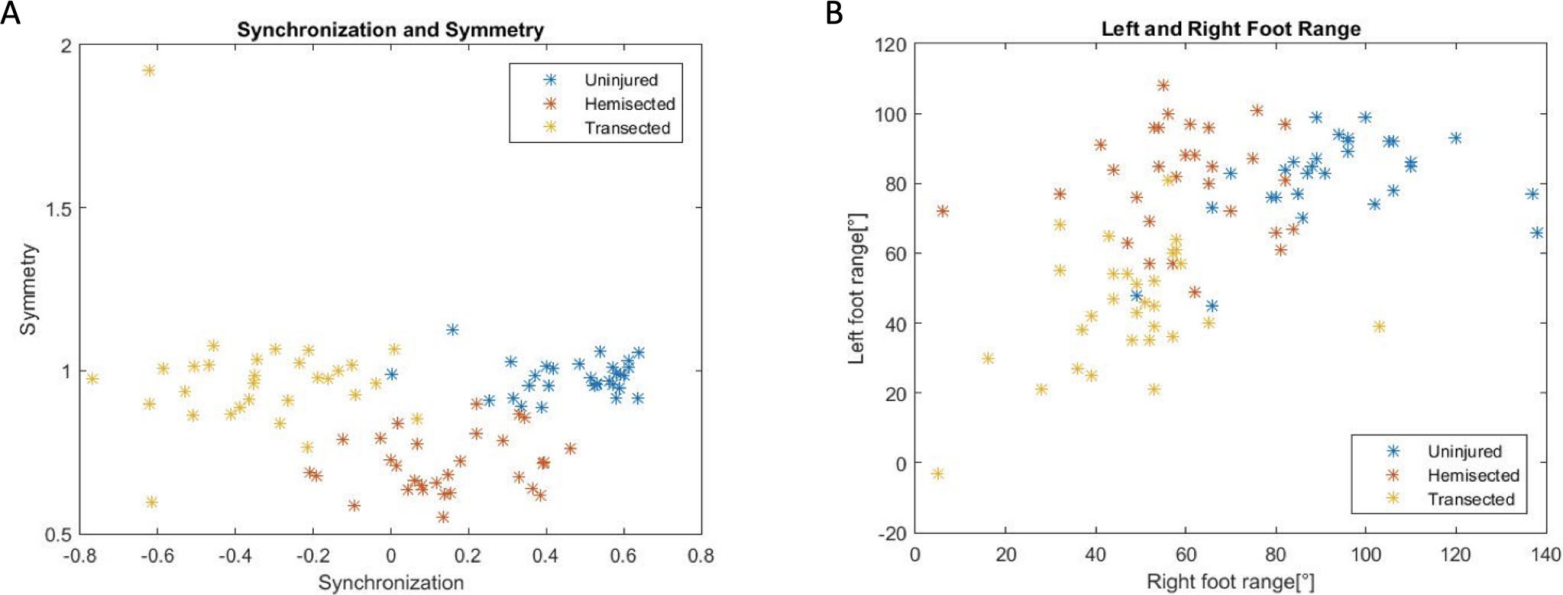
**Examples of distribution in the feature space** **of the 90 videos using two kinematic features.** They are colored with their original class (uninjured, hemisected and transected). In panel A the features synchronization (F1) and symmetry (F2) are shown, whereas in panel B features of right and left foot angles range (F3 and F4) are shown.

All results of the videos were plotted in Fig. 3A for the two kinematic features of symmetry and synchronization. As expected, healthier froglets were identified by the algorithm through their more synchronized swimming, which resulted in a positive symmetry coefficient (F1). While most transected froglets had a negative correlation of synchronization, indicating inverted movement, hemisected froglets’ synchronization results varied, with a range between −0.2 and 0.5, so not all of them were identified as having independent movement of their hindlimbs by the algorithm. However, the swimming of hemisected froglets differs from that of uninjured and transected froglets since most of them obtained a coefficient of symmetry (F2) of less than 0.9. Furthermore, both transected and healthy froglets have a similar level of symmetry in their swim, near to +1: in the case of the uninjured froglets this is explained because they make similar strokes with both left and right hindlimbs, and in the case of the transected animals it is because they have little movement in both hindlimbs. The hemisected froglets clearly show less movement in their right hindlimb in comparison to the left. This is expected, as all hemisected froglets were damaged on the right side of the spinal cord.

**Fig. 4.**
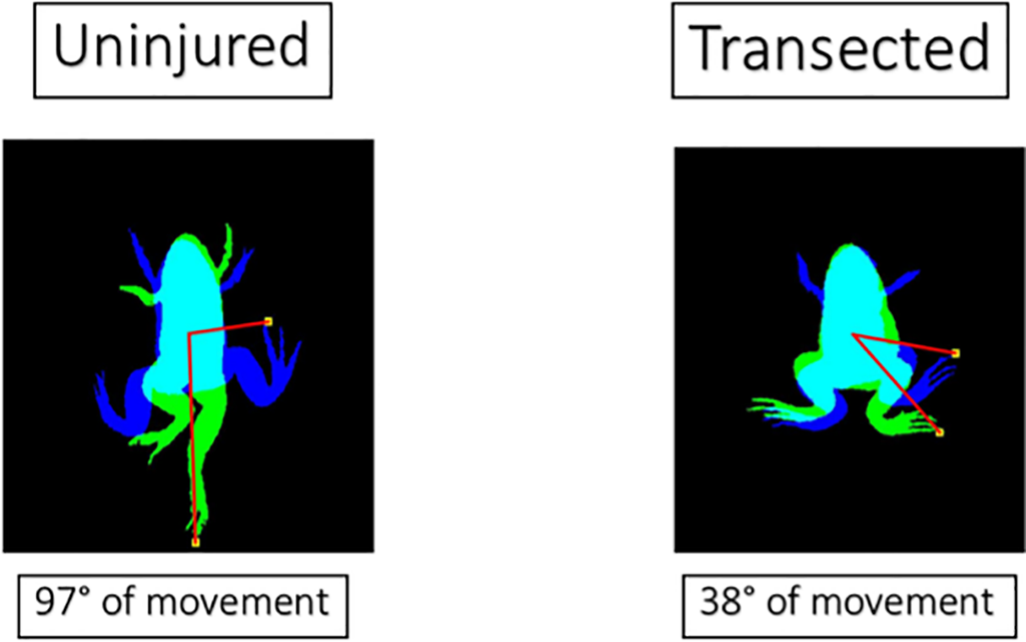
**Two examples of the right foot range (F3) measured.** The blue shadow shows the moment where the froglet has its foot in the highest position, while the green is the lowest position. The range is defined as the arc between the highest and lowest position. The healthy froglet has a relatively large range of movement while the transected one only moves it's pelvis, having a relatively small range of movement.

As expected, uninjured froglets have a relatively high range of movement in both feet, while transected froglets have a small range (Fig. 3). Hemisected froglets fall in-between, having a similar range to a healthy froglet in their left hindlimb, while the right is similar to a transected froglet. In Fig. 4 two examples are shown, where a healthy froglet reaches a 97° of movement, meanwhile a transected froglet reaches a 38° of movement. There is more confusion in the classification using range features than using synchronization and symmetry features.

### Classification results

In our experiments we tried to use both simple classifiers (e.g. minimum distance and linear discriminant analysis) and more complex ones (e.g. SVM and neural networks).

Each classification experiment was performed using cross validation with ten folds (as described in the Materials and Methods) and repeated ten times for each classifier used (Table 1). Using only two features, synchronization and symmetry, common classifiers such as k-nearest neighbors algorithm (KNN) with three and five neighbors get over 90% accuracy, which is not considerably lower than the 93.9% accuracy with a neural network. Using only the right and left foot range almost all classifiers get close to an 80% accuracy. Finally, the best result is obtained by combining all four kinematic features in an LDA resulting in a 96.6% accuracy.

**Table 1.**
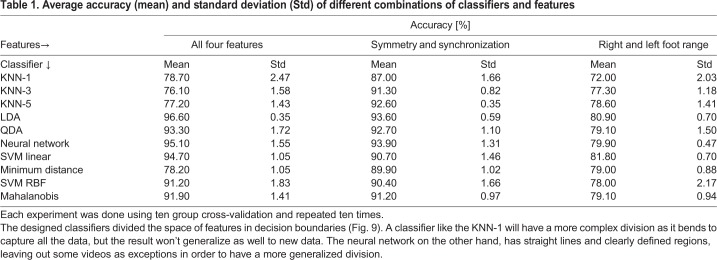
**Average accuracy (mean) and standard deviation (Std) of different combinations of classifiers and features**

## DISCUSSION

The main contributions of the paper are twofold. First, we define four kinematic features (synchronization, symmetry, range of right foot and range of left foot) that can be used to characterize the swimming behavior of the froglets. Second, we validate how relevant these features are in a classification problem, where the proposed features can be used to determine the level of the spinal cord damage of the froglets and the identification of new treatments for improving SCR.

We present a fully automatic detection algorithm that can characterize the level of spinal cord damage of a froglet's spinal cord by tracking its hindlimb movements while it swims in laboratory conditions. A 1 h processing time for every video may seem lengthy, this process is completely unsupervised, not using the researcher's time. However, the alternative of having a human segmenting and tagging each limb would be considerably slower than 1.12 s per frame. This will let researchers process larger amounts of videos without the need to manually label each limb in each frame. In addition to the detections in less time, we have also provided four kinematic features that can be used to summarize the results and variance between healthy and injured froglets. An example would be a froglet showing signs of recovery after receiving treatment. The simple setup makes it easy to reproduce the conditions of the experiment with common equipment.

Our approach has a high accuracy in classifying *Xenopus laevis* froglets in three categories of spinal cord damage using four kinematic features. It does not lose much accuracy by only using two of them (synchronization and symmetry), by being able to show the results in a 2D graph. As the proposed features create a high difference between the classes, more complex classifiers get similar results to simpler ones.

Also, this system could be used to compare the swimming of froglets where the independent variable is something other than spinal cord damage, like thermal stress (Padilla et al., 2019), genetics, etc. Our algorithm does not evaluate the z axis, making it unsuitable for a question about tridimensional behavior, which is considered as a complex behavior in terms of mobility and locomotion (Macrì et al., 2017). We did not evaluate this behavior because it induces unnecessary stress in the respiration of the lesioned froglets, which are not able to reach the surface when they need to.

It is worth mentioning that some videos fall outside of their class distribution, making them prone to being erroneously classified. These errors come from two different sources: the first one being minor errors of tracking and measurement of said kinematic features (this error is produced by our algorithm); the second source is uninjured froglets that are turning while swimming. When a froglet tries to turn to avoid the borders of the container, it uses only one of its legs to swim forward. Thus, the algorithm interprets this as independent movement. If the uninjured froglet turns for the whole video, then the algorithm will wrongly interpret said movement. This could be corrected by only analyzing frames where the froglet is swimming far from the boundaries.

The algorithm depicted here could have potential applications in screening libraries of compounds to identify new drugs that could be used to improve functional recovery after SCI. Having this algorithm should allow the measurement of small improvements in froglet swimming capacity when comparing froglets treated with those compounds with control froglets. Identifying compounds that improve SCR in froglets could be a first and easy step in selecting candidate drugs that could later be tested in more expensive model organisms, such as mouse and/or monkeys, as another step before performing clinical trials in humans.

## MATERIALS AND METHODS

We used the supervised methodology of pattern recognition (Bishop, 2006). This methodology uses the knowledge of an expert to train a model. In our case, we know how injured the froglets are and the idea is to have an algorithm based on the trained model that can predict the level of damage by analyzing a video without the intervention of the expert. A typical computer vision system based on pattern recognition follows a five-step schema: (1) image acquisition, a digital image or video of the object under test is taken (sometimes resized) and stored in a computer; (2) preprocessing, the digital image is improved in order to enhance the details; (3) segmentation, the image of the object of interest is identified and isolated from the background of the scene; (4) feature extraction/selection, significant features of the object are quantified and; (5) classification carried out by a classifier (Bishop, 2006). The five steps must be designed for a specific application using training data. In our case, the information is taken from videos of swimming froglets that are processed and segmented in order to detect their anatomy. Afterwards, certain features are extracted from the movement of the froglets, which are used to determine the spinal cord damage category.

**Fig. 5.**
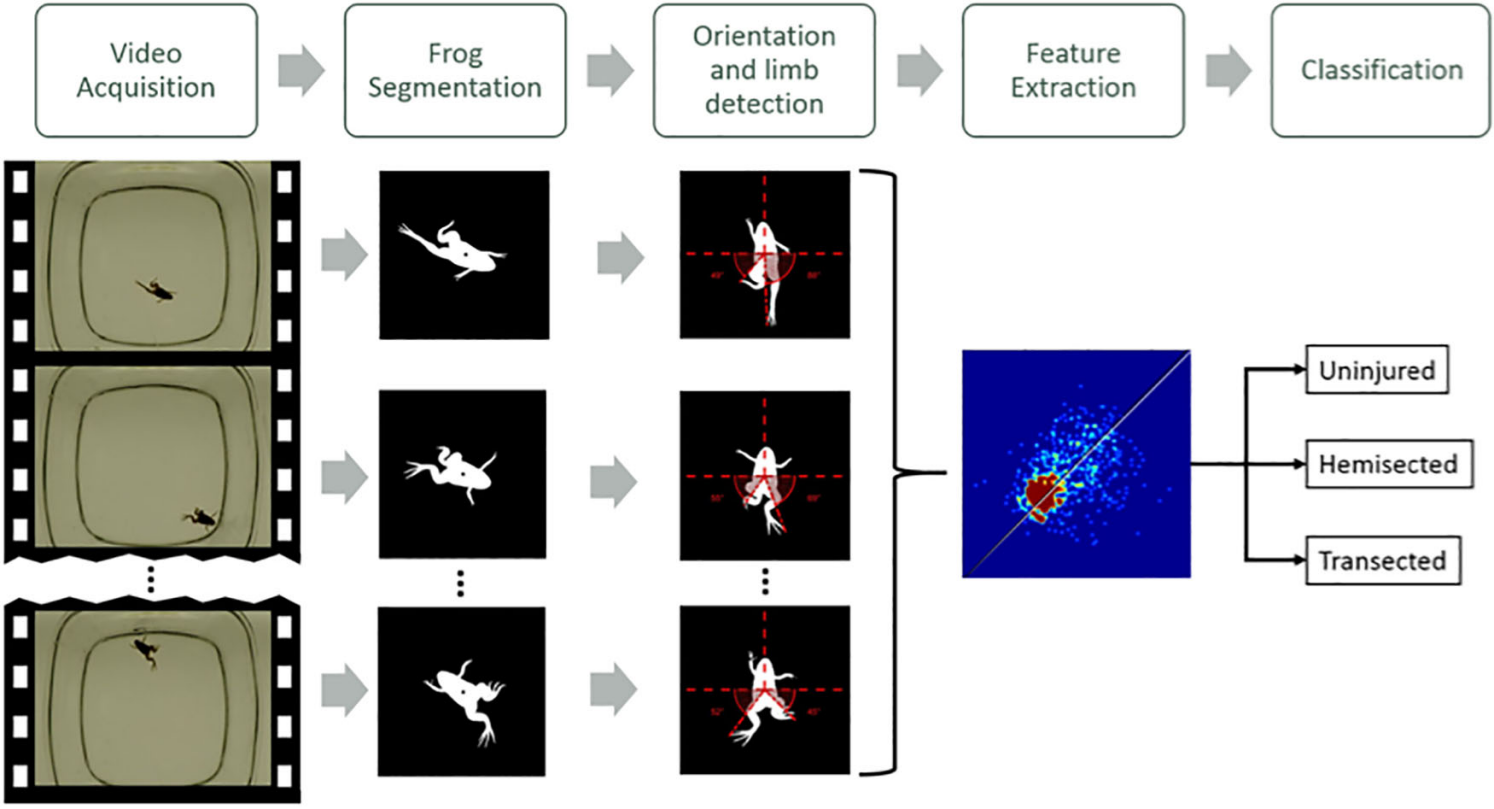
**Diagram illustrating the five step process for each video.** The whole video is processed frame by frame and then summarized in the feature extraction phase. Finally, the video is classified into one of the three levels of spinal cord damage.

Following and adapting this scheme, we have divided our algorithm into five blocks that are shown in Fig. 5. As we mentioned in the Introduction, the objective of our experiment is to correctly classify all 90 videos into the three spinal cord damage categories (uninjured, hemisected and transected). The algorithm first identifies the froglet in each frame and segments its shadow for the next step. This image is then oriented so as to always be looking upwards while in the dorsal view and detects the position of its limbs with respect to the center of gravity. The whole process outputs four kinematic features (synchronization, symmetry and range of the angles for the right and left foot) and a 2D heatmap that shows the angles of both legs of the froglet moved throughout the video. Then, a classifier is used to predict to which of the three previously defined categories of spinal cord damage the froglet belongs to. The following subsections describe in greater detail each part of the algorithm.

### Spinal cord injury of *X. laevis* froglets

**Fig. 6.**
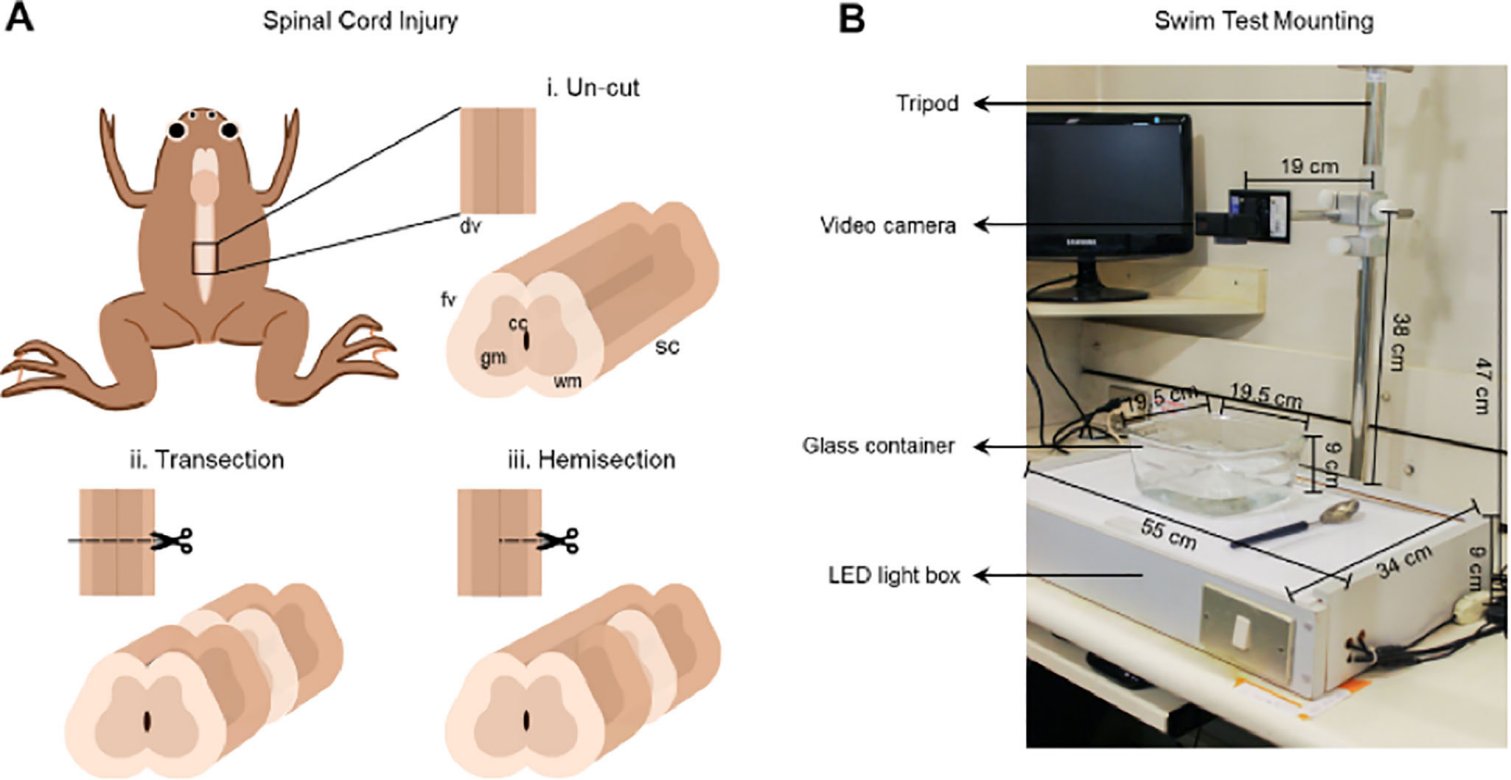
**Experimental Setup.** (A) Illustration of spinal cord injuries in the 6th vertebrae of *X. laevis* froglets in the dorsal and frontal views, (i) uninjured, (ii) transected and (iii) hemisected animals. (B) Picture of the setup used to record the movies of froglets. The tripod where the video camera is placed, a box internally illuminated with LED lights, a glass container with Barth solution and a spoon and a Pasteur pipette for swimming stimulation of froglets. fv, frontal view; dv, dorsal view of the spinal cord; sc, spinal cord; gm, grey matter; wm, white matter; cc, central canal.

*Xenopus laevis* froglets were used at 2 weeks after reaching post-metamorphic stage 66, at which point they measured approximately 1 cm wide and 1.5 cm long (Fig. 6). Spinal cord transection was performed as described in Edwards-Faret et al. (2017). Animals were anesthetized by immersing them in 0.02% MS222 0.2 g/l for 10 min and they were then placed on a gauze over an inverted petri dish to avoid any spinal cord damage. Using microdissection scissors, an incision was made at the level of the sixth vertebrae and laminectomy was performed followed by full spinal cord transection – or only the right half of the spinal cord was transected for the hemi-section. After injury, muscle and skin were put together using forceps to allow closure and healing of the incision. After surgery, both hindlimbs were paralyzed in transected froglets, whereas only the right hindlimb was paralyzed in hemisected froglets.

Careful postoperative manipulations were carried out as previously described (Edwards-Faret et al., 2017). After surgery, the froglet was maintained in the gauze and transferred to plastic tanks containing 200 ml of 0.1x Barth solution (it is not possible to specify a unit of measurement; BARTH is the standard used with frogs) with 1x antibiotics, with no more than six froglets per tank. To allow breathing, the solution did not cover the froglets' heads. The recovery of anesthesia occurred 5–10 min after the surgery, when animals began to move their upper extremities, they were then transferred to the larvae/froglet room at 20–21°C. We fed the animals 1 day after surgery with Nasco Frog Brittle pellets and the pellets were brought to the forelimbs with a Pasteur pipet to help them eat. The peeling skin was removed with forceps under a dissecting microscope, once a week. It was necessary to change the Barth x1 solution with antibiotics every other day and clean the tanks.

A total of 90 froglets were recorded, 30 froglets were used for each condition. The swimming test was performed 3 h after surgery once the animals had completely recovered from anesthesia. All experiments with froglets and the procedures used in this work have been approved by the Comité Ético Científico para el Cuidado de Animales y Ambiente at the Pontificia Universidad Católica de Chile. Number of identification: 150408002.

### Video acquisition

Uninjured, hemisected and transected froglets were recorded in the dorsal view using a GoPro Hero5 Black camera that was mounted on a tripod as illustrated in Fig. 6. Animals were maintained in a glass container with 500 ml of 0.1x BARTH solution (pH 7.6) supplemented with penicillin-streptomycin at room temperature (25°C). The froglets were placed on the glass container using a spoon and filmed one-by-one for 1 min after carefully touching the forelimbs with a Pasteur pipette (swimming stimulation). The data was acquired at 120 color frames per second with a resolution of 1920×1080 pixels for each color channel (red, green and blue). The length of the videos is approximately 1 min.

### Froglet segmentation

#### Yolo deep learning network

The first step of the segmentation is based on a deep learning algorithm called Yolo-v3 (You Only Look Once) version 3 (Redmon and Farhadi, 2018 preprint). A subset of 1800 frames were selected randomly from 30 videos from of our dataset (ten videos of each spinal cord damage category). In addition, we manually detected the froglets in each video in order to generate the training dataset. The model was trained for 2000 iterations and validated visually with images from the other 60 videos.

#### Area range calibration

During the identification and tracking, the algorithm filters out objects that have an area too large or too small to be the froglet (to prevent the detection of other objects located in the video). In order to do this, the area of the froglet is measured. For each video six random frames are extracted, the froglet is then detected in each image using the Yolo Network previously described. By using a color threshold and the x, y coordinates of the detection in the frame, the area of the froglet is measured in each of the frames of the video. The calibration area is defined as the median of the measured areas of the images. Then the froglet's area range is defined as 10% larger and smaller than the calibration area. Multiple thresholds were tested using our video database and 10% was observed as difference of area of the froglet throughout a video.

#### Froglet detection

The algorithm processes one every two frames to reduce processing time (in our experiments, this rate still maintained accurate results), while maintaining accurate results. The first step was a threshold in the blue channel, which of the three color channels has a larger difference between the froglet and the background creating a better segmentation. This outputs a binary image where the froglet was seen as white and the background as black. The area of each object in the image was measured, filtering those whose area fell outside the range defined in the calibration section. Of the remaining objects, the one closest to the previous frame detection is chosen as the froglet. A fixed size window of the frame is extracted, the coordinates of the center of the frame are used for the detection of the next analyzed frame.

### Orientation and limb detection

#### Orientation

The binary segmented image is virtually eroded in order to eliminate the limbs, leaving only the body. Virtual erosion is accomplished by thinning the outer pixels of the object in the binary image. The orientation is measured using the Matlab function ‘Regionprops: Orientation’ (MathWorks, 2017). The image is then oriented so as to always be looking upwards while in the dorsal view and detects the position of its limbs with respect to the center of gravity. By checking the number of pixels in the bottom half versus the top half of the image (because of a difference of pixels between both halves) one can correct that the froglet is ‘looking up’ and not ‘looking down’.

#### Limb detection

**Fig. 7.**
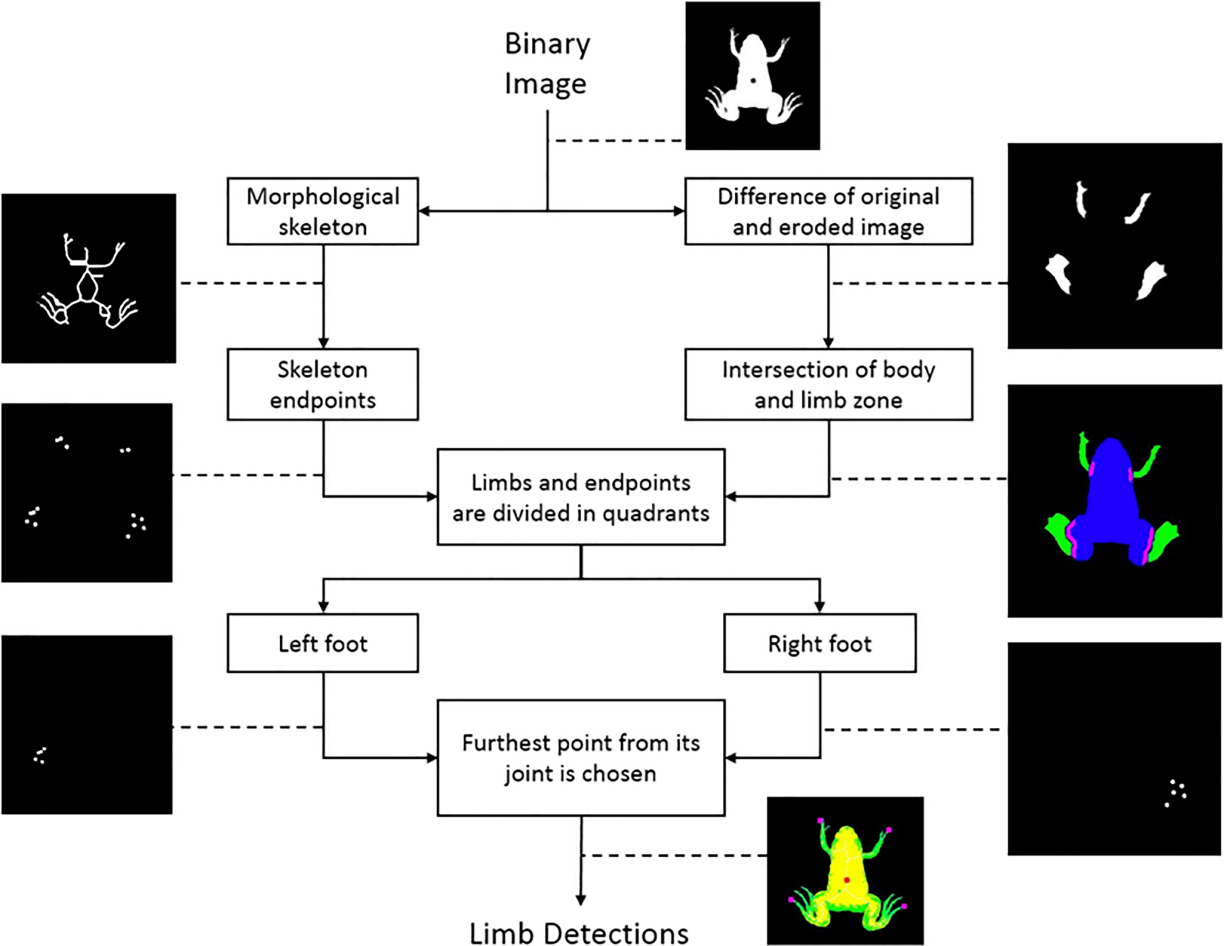
**Diagram showing the steps to detect the froglet’s limbs.** The algorithm detects limb zones, joint zones and endpoints for each of the four quadrants. Each limb is defined by the endpoint furthest from its joint.

The limb detection algorithm (Fig. 7) starts with a binary image of the previous step. By subtracting an eroded image from the original one, the limbs are extracted. Both limbs and body of the froglet are then dilated to find the area where they intersect. The dilation adds pixels to the boundaries. This area is defined as the ‘joint’ of limbs and body. Using Matlab function bwmorph: skel the ‘morphological skeleton’ is extracted from the segmented image. Using bwmorph: endpoints, the endpoints of the skeleton are extracted. These ‘points of interest’ are considered as the hands or feet of the froglet. The information of both branches is combined and divided into four quadrants, one for each limb. For each quadrant the furthest point of interest from its joint is selected as the foot or hand of the froglet.

**Fig. 8.**
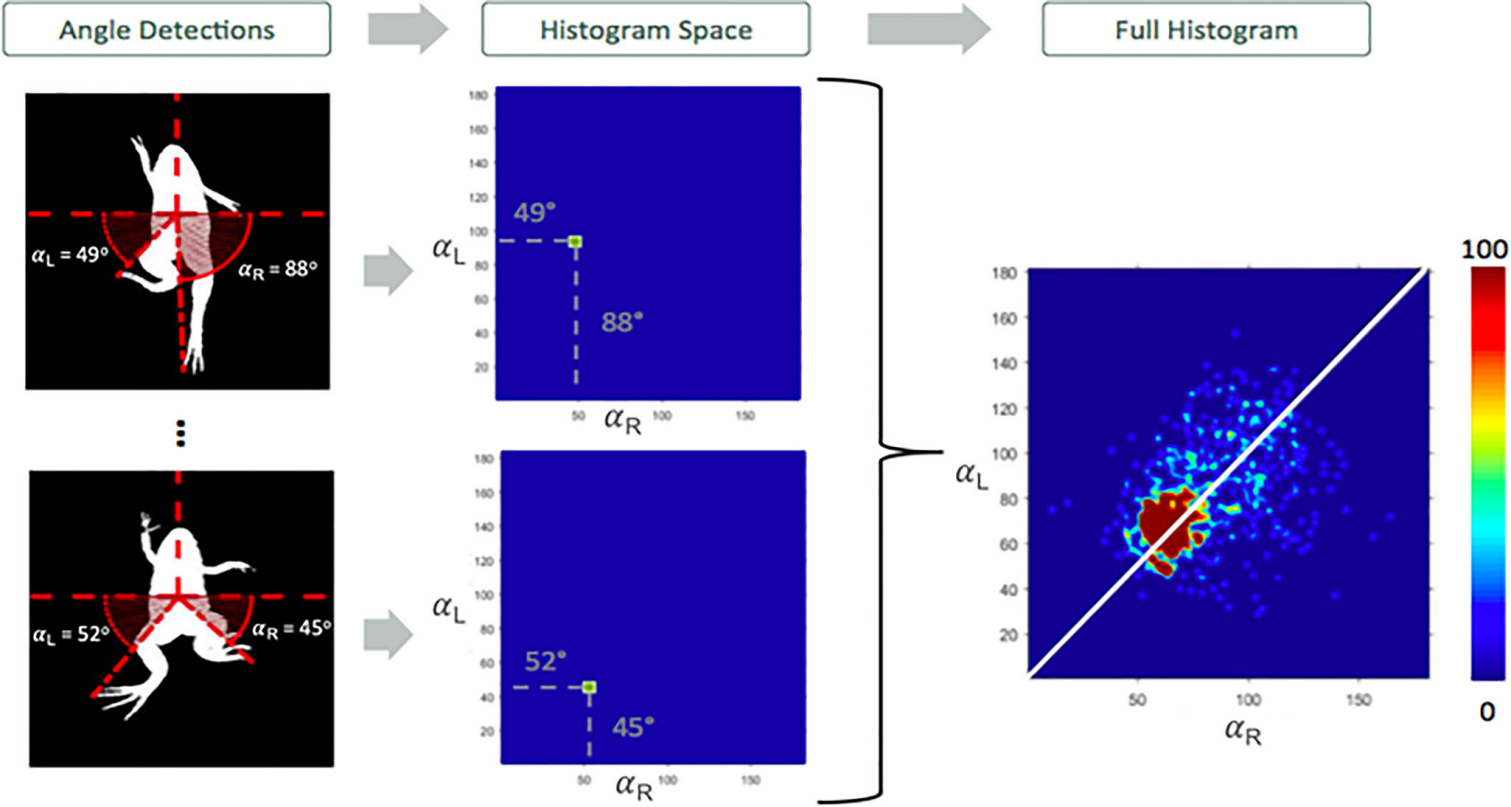
**Example of a 2D histogram is constructed and outputted by the algorithm.** In the examples, the angle-pairs (*α*_R_, *α*_*L*_) are 49°, 88° and 52°, 45°. For these frames, the bins – 49, 88 and 52, 45 – of the 2D histogram are increased by one. The histogram is represented on the right as a heatmap with a color scale; the deepest red value is associated with the most common angle-pair observed throughout the video, while the blue is associated with an angle-pair not seen during the video. The white line represents perfect symmetry, meaning that both feet are horizontally mirroring.

After the limbs are detected in a frame, the angle-pair of the limbs (*α*_R_,*α*_L_) is computed (Fig. 8). The angle-pair is defined as the two angles between the right and left foot and the horizontal line. This makes it easier to compare the movement of both legs. We define a 2D histogram as a matrix of 180×180 bins, where each axis represents the angles *α*_R_ and *α*_L_, and each bin (with a resolution 1°×1°) counts how many frames of the video have the legs in the angle-pair defined by the bin. To show which angle-pairs were more common to the froglet during the video, the histogram is normalized between the maximum and minimum bin value and visualized as a heatmap. The most common angle-pairs are shown with a red color, while a non-detected angle-pair throughout the video is colored blue.

### Feature extraction

Information from the videos are summarized in four kinematic features. The contributions of these features are threefold: (1) they are highly discriminative, that means in this classification problem they achieve a high level of variance between classes and maintaining a low level of variance within a class. (2) They are derived from expected visual behavior with an easy interpretation. (3) They can be easily computed using a mathematical formula. In addition, these features allow a rapid understanding of which parameters of froglet swimming are altered and which can be improved with different treatments.

#### Synchronization

The first feature referred to the synchronization of hindlimb movement. It captures the synchronization of the feet while swimming. Three types of synchronization are presented to illustrate the observable behaviors: (1) ‘synchronized’, fore- and hindlimbs from the right and left move forward or backwards at the same time. (2) ‘Independent’, only one of the hindlimbs moves while the other stays still. (3) ‘Inverted’, one hindlimb moves forward while the other moves backwards and vice versa. By observation, the healthy froglet should swim synchronized, while a hemisected froglet should move only one hindlimb and a transected froglet cannot move both hindlimbs, but the movement of the pelvis is identified as an inverted movement of the feet by the algorithm.

Synchronization was measured as the correlation between the angles *α*_R_ and *α*_L_ (Fig. 8). If the correlation between the feet was close to 1 then we determined that both feet were synchronized in their swimming. On the other hand, if the correlation was close to −1, the movement of the feet was inverted. And, if the correlation was close to 0 then the feet were moving independently of each other, which potentially means the froglet is moving one foot while leaving the other still. As the froglet will have slightly unsynchronized movements, the expected synchronization coefficient for synchronized swimming will be closer to 0.5, while an inverted movement will be close to −0.5.

#### Symmetry

The second feature is symmetry in the feet's strokes. In the average stroke of a healthy froglet, we observe that both feet should move in a similar arc. Alternatively, a hemisected froglet will move the healthy foot in a larger arc than the damaged side. In our observations, the transected froglet has little movement of its legs, but said movement will tend to be symmetrical.

Stroke symmetry can be measured with a first order linear regression. The linear regression finds out the relationship between the angles *α*_R_ and *α*_L_ by finding the slope β which best solves the equation:
(1)
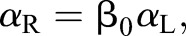
where β is the symmetry coefficient. If both feet move symmetrical to each other, β₀ will be close to 1, but if the right foot moves half of the arc of the left foot, then β will be close to 0.5.

#### Range

The last two features are the range of angles *α*_R_ and *α*_L_. A healthy froglet has a relatively larger range of angles of both of its legs, while a transected one will have a relatively smaller range. The non-injured leg of a hemisected froglet will fall somewhere in-between, having more movement in its uninjured side than the damaged side. To measure this, the detections of each foot are smoothed using a bivariate kernel density estimator (KDE) and a Gaussian convolution (Botev et al., 2010), which is a good solution for noise reduction. A threshold is applied to the result and an area of movement of the foot is obtained. The highest and lowest point is calculated from that area and the range of movement is the arc made by both points and the center of gravity of the froglet.

### Classification

**Fig. 9.**
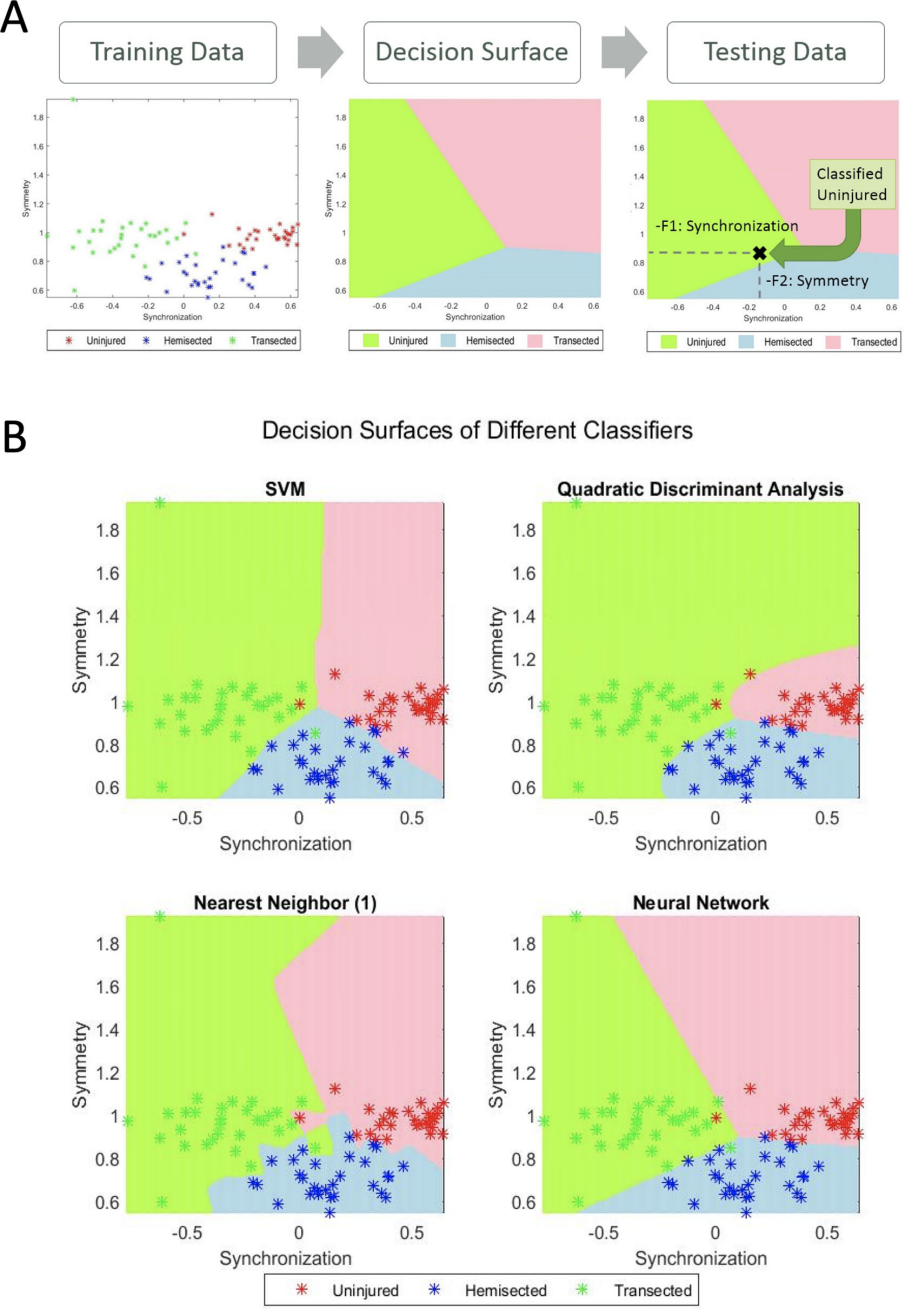
**Examples of the output of a classifier plotting** **all 90 videos using two features: synchronization (F1) and symmetry (F2).** (A) Diagram of a classification process. Labeled training data is computed to a decision surface of the feature space. This is a mathematical formula that depends on the type of classifier. New data can be tested and classified depending where it falls in the decision surface. (B) Different classifiers and how they divide the feature space in decision boundaries.

The final step of the algorithm is a classifier. In our case, the idea of the classifier is to assign the froglets to one of the three classes: uninjured, hemisected and transected. The classifier takes the extracted features of a froglet and assigns the froglet to one of these classes. The extracted features are arranged in a vector of n elements that are represented in a feature space of dimension n. For example, if we extract the features F1 (synchronization) and F2 (symmetry), the feature space is a 2D space, where x, y axes correspond to the variables F1 and F2, respectively. Thus, a froglet was represented as a point in this 2D space as illustrated in Fig. 9. In order to design a classifier, the feature space is divided into three regions, which we called ‘decision regions’, one for each class. This step is called training, in which a model that finds the decision regions is estimated. After training if we want to classify a new froglet we extract the proper feature vector, a point in feature space, and classify it according to the decision region to which the point belongs.

It is worth mentioning that when designing a classifier, two stages are taken into account: training and testing. On the one hand, in the training stage, training data (froglets for training purposes) are used to define the decision regions. Typically, a classifier defines the decision regions according to the concept of similarity: features that are similar are assigned to the same class. On the other hand, in the testing stage, testing data (other froglets that were not used in the training stage) were used to evaluate the performance of the classifier. That means features were extracted from the testing froglets and classified using the trained model. In the evaluation, the accuracy defined as the ratio of test froglets correctly classified is computed. In our experiments, we tested simple classifiers because of the small number of samples that were available. The classifiers that we used were: minimum distance, linear discriminant analysis (LDA), k-nearest neighbors (KNN) with 1, 3, 5 neighbors; and more complex ones such as: quadratic discriminant analysis (QDA), a shallow neural network, Mahalanobis distance and support vector machine (SVM) in both its linear and radial basis function kernel (RBF) (Bishop, 2006). To this end, we used the implementation of Balu Matlab Toolbox (Mery, 2011).

The performance of the classification was evaluated using cross-validation (Mitchell, 1997). In our experiments, the data are divided into ten folds (independent groups), because it has become the standard method in practical terms (Witten and Frank, 2005). That means 90% were used for training (81 frogs) and 10% for testing (nine frogs). This experiment was repeated ten times, interchanging training and testing data to evaluate the stability of the classifier. Then, when training was performed, the samples that were initially removed could be used to test the performance of the classifier on these test data. For each test, the performance defined as the rate of samples correctly classified is computed as η_i_, for i=1…10. Thus, we evaluated the generalization capabilities of the classifier by testing how well the method classified samples that had not been already examined. The estimated accuracy, η, is calculated as the mean of the ten percentages of the true classifications that are tabulated in each case: η=(η_1_+…+ η_10_)/10.

To validate both the algorithm and kinematic features, the performance of different pattern recognition classifiers was measured to see how much variance exists between the three categories of spinal cord damage. A high accuracy of classification shows that the proposed system of algorithm and features can be used to differentiate levels of spinal cord damage, which is useful in comparing damage levels in different froglets.

### Implementation

The algorithm was implemented in Matlab except for the Yolo approach, which was done using the original implementation given by the author (Redmon and Farhadi, 2018 preprint). The average processing time was 1.12 s/frame in a 3.4 GHz Intel i7 processor, which takes about 1 h of processing for each 1-min video. The output of our code is a list of the detection information for each frame, the classification of the froglet (one of the three spinal cord damage categories) and the four kinematic features that can be used to further study the swimming performance. Code and videos are available (see https://domingomery.ing.puc.cl/material/).
